# Leading through expertise: a qualitative study of clinicians' experience of a paediatric clinical trial for displaced medial epicondyle fracture

**DOI:** 10.1302/2633-1462.69.BJO-2024-0258.R1

**Published:** 2025-09-11

**Authors:** Elizabeth Tutton, Emma E. Phelps, Janis Baird, Matthew L. Costa, Juul Achten, Amy Moscrop, Phoebe Gibson, Daniel C. Perry

**Affiliations:** 1 Kadoorie, Oxford Trauma and Emergency Care, Nuffield Department of Orthopaedics, Rheumatology and Musculoskeletal Sciences, Oxford University, Oxford, UK; 2 Major Trauma Centre, Oxford University Hospital NHS Foundation Trust, John Radcliffe Hospital, Oxford, UK; 3 MRC Lifecourse Epidemiology centre, University of Southampton, Southampton, UK; 4 Alder Hey Children’s NHS Foundation Trust, Liverpool, UK; 5 Department of Child Health, University of Liverpool, Alder Hey Hospital, Liverpool, UK

**Keywords:** Qualitative, Paediatric, Trauma, Trial, medial epicondyle fractures, clinical trials, randomized controlled trials, Clinicians, nonoperative treatment, paediatric orthopaedic, orthopaedic injury, orthopaedic surgeons, COVID-19 pandemic, EpicoNdyle

## Abstract

**Aims:**

We sought to explore staff experience of a paediatric randomized controlled trial (RCT), comparing operative fixation and nonoperative treatment for displaced medial epicondyle fractures.

**Methods:**

A total of 20 staff (eight surgeons and 12 research delivery staff) recruiting to the RCT in 18 NHS Trusts across the UK took part in a telephone/online qualitative interview. Interviews were informed by Heideggerian Phenomenology and thematic analysis.

**Results:**

We identified the concept of 'leading through expertise' demonstrated through two themes: 1) choosing ways of making it work; and 2) enabling parental/child decision making. Staff drew on their clinical and organizational expertise to take a position of equipoise and invested time to make the trial work within their local context. Building trust and confidence and using creative ways to engage with children enabled parent/child decision making in the context of uncertainty. Recruitment was sustained by the energy, enthusiasm, and expertise of staff, by the local investigator and research delivery staff, the digital resources, and the support of the trial team. Recruitment was hindered by clinical pressures, a variable research culture, and lack of consistent digital access.

**Conclusion:**

For this relatively rare injury in children, ‘leadership through expertise’ was critical for successful trial recruitment. National and local networks of surgeons were imperative to support the trial activity. The development of similar networks among research delivery staff could improve knowledge exchange and enhance trial activities. Parental/child trust and confidence to decide about trial participation could be enabled by work to deepen child engagement in research. Educational tools engaging children may help to support family decision making in an emergency context.

Cite this article: *Bone Jt Open* 2025;6(9):1090–1100.

## Introduction

The treatment of medial epicondyle fractures is one of the biggest ongoing debates among orthopaedic surgeons treating children. Some argue that the bone fragment should be surgically reattached, restoring the anatomy including the soft-tissue attachments. However, others argue that there is no benefit in surgical reattachment, which would unnecessarily expose the child to the risks of surgery and lifelong scar. Consequently, the British Society for Children’s Orthopaedic Surgery has identified this as a top priority for further research.^1^ To address this uncertainty, surgeons throughout the UK, New Zealand, and Australia recruited to a randomized controlled, multicentre, prospective superiority trial of operative fixation compared with nonoperative treatment for displaced medial epicondyle fractures: the Surgery or Casts for Injuries of the EpicoNdyle in Children’s Elbows (SCIENCE) study.^2^

Recruitment to randomized controlled trials (RCTs) can be challenging, which is amplified among child participants, and further amplified with a significant difference between treatment interventions (i.e. surgery or no surgery). However, there is limited research that examines staff experience of recruiting to studies in paediatric orthopaedic trauma. In adult orthopaedic trauma, recruitment is enhanced by strong clinical and research teams where staff have equipoise and the ability to integrate the study into everyday clinical practice.^3^ In rare, complex orthopaedic injury an illusion of equipoise can exist, though recruitment cannot be sustained.^4,5^ In paediatric orthopaedic trauma, the CRAFFT study^6^ of surgical reduction and non-surgical casting for distal radius fractures identified difficulties among staff with equipoise, the eligibility criteria, integrating the study into practice, interdisciplinary working, and parental preferences for treatment. Nevertheless, the clinical community overcame these challenges to successfully recruit to target within the CRAFFT study.

Understanding of children’s experience of trial recruitment and recovery after injury is limited. Children interviewed in the SCIENCE study indicated they have similar concerns to their parents. They were uncertain about the trial treatments and wanted advice on pain management, recovery, and return to normal activity and sports.^7^ Parents in the CRAFFT study worried about reinjury and noted differing perceptions between themselves and their child.^8,9^ Staff views of how they engage with children about this trial will build on existing qualitative findings from the SCIENCE and CRAFFT studies. This study therefore aimed to explore staff experience of what helps and hinders recruitment in emergency care of a rare paediatric injury through the SCIENCE study.

## Methods

This is a qualitative evaluation, embedded within the SCIENCE study,^2^ a randomized controlled, multicentre, prospective superiority trial of operative fixation compared with nonoperative treatment for displaced medial epicondyle fractures recruiting throughout the UK, New Zealand, and Australia.

The study drew on Heideggerian Phenomenology,^10^ an approach used in studies of traumatic orthopaedic injury to explore how participants know and understand their world in the context of their everyday clinical practice.^11,12^ The research explored the reality of participants’ experience, their understandings, thoughts, feelings, and relationships within the context in which they worked. Temporality was also important as experience changes over time. The process of analysis was interpretive and required reflection on elements of experience to understand the collective experience of the group of participants. In-depth qualitative interviews focused on what it was like to be part of the SCIENCE study, what was important, and what was taken for granted? Interview prompts were: What did you think? How did you feel? Tell me more about that. Questions were iterative to expand developing categories and themes. Interviews were audio recorded and transcribed verbatim. Participants received a participant information sheet detailing the study and had at least 24 hours to consider their participation. Informed consent was verbally recorded prior to interview. Ethical permission for the study was included within the main SCIENCE study (19/NW/0158, 25 March 2019). The trial is registered through the ISRCTN registry (16619778: 18/03/2019) and the protocol is published online.^2^

### Sample

A purposive sample of 20 staff, eight surgeons (seven male), and 12 research associates (nurses and other health professionals involved in research delivery, one male) from 18 NHS trusts in England, Wales, and Northern Ireland took part in interviews. In total, 16 sites were teaching hospitals and two district hospitals, recruitment to the SCIENCE study ranged from one to 29 participants per site (mean 9). The term research associate encompasses any staff involved in the delivery of the study and is used to ensure anonymity when describing healthcare professional groups with few participants. One person declined to take part due to work pressures. The study was disrupted by the COVID-19 pandemic and interviews took place between November 2019 and December 2021, and August 2022 and October 2022. The duration of the interviews was 20 to 74 minutes (mean 40 minutes).

### Patient and public involvement and engagement

Two parent co-investigators (AM, PG) have been part of the SCIENCE study conception, design, and study materials, trial management, and steering committees. They engaged with the analysis of the qualitative data, are co-authors, and have been integral to the development of this article.

### Analysis

Data were coded line-by-line. Codes were brought together to develop categories and then themes. Similarities and differences within themes and across the dataset were examined. For example, the codes ‘avoid treatment decisions prior to providing study information’ and ‘staff direct patients to my clinic’, were coded in the sub-category ‘teamwork’. Teamwork was linked with other similar sub-categories to create the category ‘taking a position of equipoise’ within the theme ‘choosing ways of making it work’. NVIVO v. 12 (QRS, UK) was used to help organize the data. Data saturation, where no new evidence is identified in categories and themes, was achieved. The researcher (ET) was experienced in qualitative health services research, considered their positionality, and engaged with another researcher (EEM), the study team and parent co-investigators during interpretation of the findings. Rigour was demonstrated through trustworthiness. Reflection on interpretation, a clear audit trail, and information about the context and method was provided to aid decisions about transferability of the findings to other settings.^13^ The study was informed by the consolidated criteria for reporting qualitative research guidelines.^14^

## Results

**Fig. 1:**
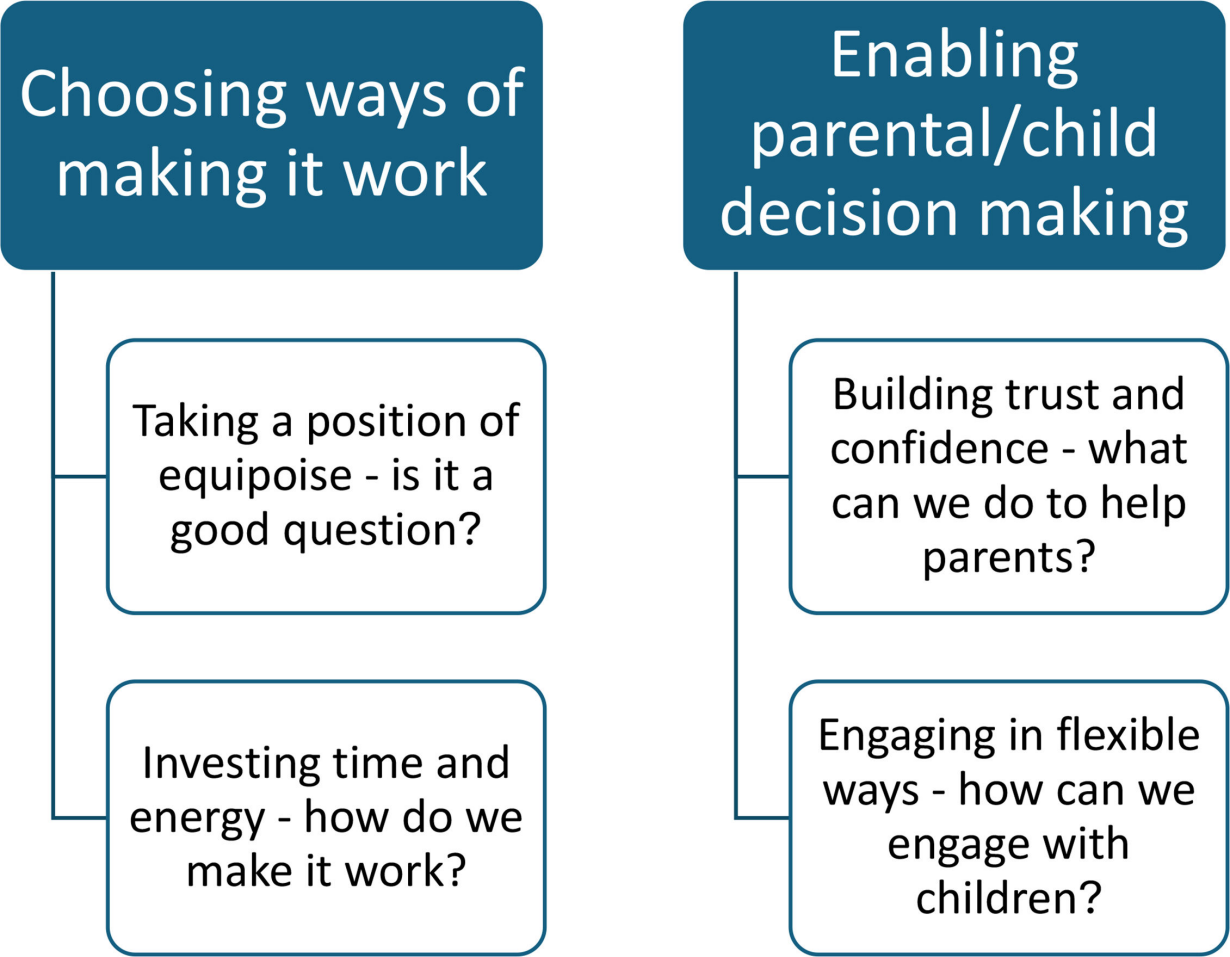
A diagram to show the two themes, each with two categories, for the concept 'leading through expertise'.

The concept of 'leading through expertise' was expressed through two themes, each with two categories (Figure 1, Table I). These were:

- **Theme 1:** Choosing ways of making it work.
  i) Taking a position of equipoise - is it a good question?
  ii) Investing time and energy - how do we make it work?
- **Theme 2:** Enabling parental/child decision making.
  i) Building trust and confidence - what can we do to help parents?
  ii) Engaging in flexible ways - how can we engage with children?

**Table I.** The two themes, each with two categories, and their definitions for the concept 'leading through expertise'.

Leading through expertise	
**Theme one**	**Theme two**
**Choosing ways of making it work**	**Enabling parental/child decision making**
Staff choose to take a position of equipoise and invest their time and energy in the study. This process was facilitated by expertise in surgical and research delivery staff, teamwork within and across specialities, digital trial design, and accessible trial support.	Staff understood the challenging nature of the decision facing parents and children when offered the opportunity to participate in a trial. Family-centred practice and engaging with children in a variety of ways, with due consideration of their distress and maturity, were valued.
**Category and definition**	**Category and definition**
**i) Taking a position of equipoise - is it a good question?**	**i) Building trust and confidence - what can we do to help parents?**
Despite personal preferences, surgeons took a position of equipoise based on the value they and national/international surgeons placed on the research question. Reflection on treatment outcomes and concerns continued as staff worked together with colleagues to recruit to the study.	Staff engendered family trust and confidence through: understanding the parent/child experience, generating interest in the research study, sharing information and supporting the family as they made their decision. Parents’ confidence to make a decision was challenged by the emergency context, the different nature of the treatments (surgery or no surgery), and treatment uncertainty.
**ii) Investing time and energy - how do we make it work?**	**ii) Engaging in flexible ways - how can we engage with children?**
Surgical staff and research associates invested time and energy integrating SCIENCE into daily practice so that the study worked in their clinical area. Staff found creating space, giving time, and developing a research pathway were key to recruitment. Creating space for research within busy clinical areas was challenging. Organizational knowledge, good relationships, dedicated research delivery expertise, and digital support facilitated this work.	Staff valued the child’s contribution within the principles of family-centred care and aimed to see family members together. They worked with families exploring preferred treatments, fears about surgery, and children’s thoughts and feelings about trial participation. A range of ways were used to communicate with children such as, play, drawing, and digital media using the tablet computer.

### Theme one

**Choosing ways of making it work:** Staff chose to take a position of equipoise and invest their time and energy in the study. This process was facilitated by expertise in surgical and research delivery staff, teamwork within and across specialities, the digital trial design, and accessible trial support.

**i. Taking a position of equipoise: is it a good question?**

Surgeons chose to lead recruitment for SCIENCE based on their surgical expertise in treating paediatric medial epicondyle fractures and the value they and their colleagues placed on the research question.

"I’d say that it’s a really good trial, it’s a good question because there is no true evidence as to which treatment method is best. For those borderline medial epicondyle fractures, which fit into the inclusion criteria, the best treatment is randomization. It’s a fair trial to do for the parents, for the patients, and for the surgeon; I think it is a spot-on trial." *S5 Surgeon*

Strong surgical leadership through taking a position of equipoise was essential. Surgeons had strong treatment preferences and experience of treating symptoms after both treatments.

"I’ve been a children’s orthopaedic surgeon for 25 years and you see good and bad results with both methods. I was basically a fixer, but I’ve got patients of my own who remained very stiff, never really completely unstiffened, and had a lot of symptoms from the screws. I’ve done a late fixation of the epicondyle in the last couple of years, which was tricky. There is plenty to be in equipoise over." *S3 Surgeon*

Taking a position of equipoise did not preclude ongoing reflection on the best treatment, particularly for minimal or extremely displaced medial epicondyle fractures.

"There was a fracture that was on the WhatsApp group [a secure end-to-end messaging service] the other day and it was wildly displaced. I wonder, if that had been randomized to nonoperative, how I would have managed that. I suppose that’s part of the deal, but it did look pretty impressively displaced. I was secretly hoping that it was randomized to fix. The completely undisplaced or minimally displaced one, if randomized to operative, you don’t think they need an operation, you’re just decorating the bone. This study will be most important for injuries where it is hugely displaced, and where it doesn’t get an operation." *S7, Surgeon*

Surgeons and research associates recruited well by working as a team, despite varying views on equipoise held by individual team members. Active collegial networks, facilitated by local and nationwide WhatsApp groups, were used to maintain visibility of the study and for practical purposes. They were used to alert colleagues when a patient was eligible, share images, study updates, and experience of treatment and outcomes. For a rare injury recruiting over a prolonged period (due to the COVID-19 pandemic), this interaction helped to sustain enthusiasm for the trial.

**ii. Investing time and energy: how do we make it work?**

Surgeons and research associates invested time and energy integrating SCIENCE into daily practice so that the study worked in their clinical area. Staff found creating space, giving time, and developing a research pathway were key to recruitment. Creating space for research within busy clinical areas was challenging.

"When we put the patients in clinic, each of them is booked in the room for a maximum of 15 minutes. Staff or consultants are reluctant to contact you to recruit patients because you’re going to be there for at least 40 minutes. I’ve timed myself and the quickest that I’ve recruited a patient is about 40 minutes." *S14, Research Associate*

Where possible, recruitment in a space away from the clinical setting or at home to reduce pressure on clinical staff, parents, and children worked well. Some parents provided virtual consent after considering the study at home. Giving time was necessary as parents and children were distressed and, in the context of uncertainty, had difficulty making an informed decision about trial participation. Time helped them process the information and staff were invested in ensuring the decision was not constrained by other clinical and research commitments.

"They do get a lot of time, and they really do need a lot of time to go through each and every bit of information." *S8, Research Associate*

Staff developed a research pathway taking into account availability of staff, expertise, and surgical willingness to take a position of equipoise. In large trauma units maintaining visibility was crucial. Smaller teams referred the patients with medial epicondyle fractures to one or a few surgeons who provided oversight throughout treatment and recovery. A high personal investment of time and energy facilitated recruitment.

"I could not [bear to] miss two patients - because of a holiday flight - so I did it on the phone and the parents were delighted. They were at home. They were getting a call from the consultant - who seems to know what he’s talking about - and then go through the whole process." *S20, Surgeon*

Research associates enabled teamwork by using organizational knowledge to develop relationships across clinical teams. This allowed them to establish and facilitate all aspects of the research process. Working as a team, around existing clinical commitments, to support families worked well.

"It was me [research associate] that was there throughout the process and the surgeon came in beforehand and at the point of randomization. We made sure that the patient was first on the [clinic] list because the orthopaedic clinics are notoriously busy. He [surgeon] obviously hadn’t got the time to be able to sit down and speak more at length with the family. So, I arrived half an hour before their appointment to speak to the family about it and he came in for parts of the process." S*1, Research Associate*

Practical elements within the study design helped to make the study easier for staff. The digital study design, digital study information, supportive trial teams, and study tablet computer were identified as valuable resources that facilitated the study.

"The information sheet is brilliant and the videos that they can watch are fantastic and of all the studies I work on I think the children’s studies are the best set up; they’re the best user-friendly and patient-friendly that I work on." *S12, Research Associate*

The large colourful tablet computer holder helped engage the family in the study. The tablet computer was used to access the website and to sign the consent/assent forms.

"Everybody comments on the size of the ipad [tablet computer[ and that’s helped; they’re like wow!" *S7, Surgeon*

Staff were challenged by some families and staff who were reticent about involvement in research activities. Early treatment decisions in emergency care could mean families had a strong preference for treatment. Variable Wi-Fi provision or incompatibility of the tablet computer with the digital provision could cause frustration.

"I was consenting a patient, and it took me about an hour and a half to do the consent because we couldn’t get the consent to load up - which you rely on for these studies." *S18, Research Associate*

Early concerns about the change from paper to digital design faded as the trial progressed but some staff had to print digital consent forms for medical notes that were not digitalized. Table II and Table III gives further supporting evidence for theme 1.

**Table II.** Examples of participants' quotes for theme one, ‘choosing ways of making it work’.

**Theme one, 'Choosing ways of making it work'** Staff choose to take a position of equipoise and invest their time and energy in the study. This process was facilitated by expertise in surgical and research delivery staff, teamwork within and across specialities, digital trial design, and accessible trial support.	
**Category and definition**	**Examples of participants’ quotes with the codes in brackets**
**i) Taking a position of equipoise - is it a good question?** Despite personal preferences, surgeons took a position of equipoise based on the value they and national/international surgeons placed on the research question. Reflection on treatment outcomes and concerns continued as staff worked together with colleagues to recruit to the study.	"From my point of view, I’m heavily supportive of the trial, no matter what my own personal belief about the treatment is. I think the challenge, from my personal point of view, has been that we should be treating them conservatively. So, when your own personal view is quite heavily in favour of nonoperative treatment, it feels harder to have that discussion, and then recruit a patient when you may end up giving them operative treatment. Which in my own mind, I don’t really believe is what I would want for my child." S13, Surgeon (Personal view) "If the child is under my care and they get randomized to being fixed, it’s not what I normally do, but I’m very happy to go ahead and do it because there is a valid clinical question. I’m happy with knowing that there’s a split in opinion, knowing that there is central equipoise and that it’s valid, even though my personal equipoise is not necessarily there. I’m very happy to run with the simple thing knowing that people have an opposite view to me, which is great - a perfect study." S16, Surgeon (Valid question supported by community equipoise) "With SCIENCE, the first patient who was managed in the nonoperative arm, at six weeks had x-rays that looked like they had not had a fracture. You could actually see some sclerosis where the apophysis had come off but you couldn’t see if the epicondyle had come off. You couldn’t see displacement, so yes, I’m very happy for the medial epicondyle to be managed nonoperatively. I think the study will be good because it will show us what is the best answer across the board. Are there some that, unlike the one I saw, are wildly displaced even after a long period of time with nonoperative management." *Participant 15, Surgeon* (The best answer) "If you can sell it enough and you get a quorum of consultants who agree to it, then the trial goes ahead and the unit takes it on. You don’t have to have every consultant on board with it; some consultants can opt out, because we can’t force people to take part in trials. What I tend to find is that once a trial has started, the other consultants who may have been ‘sat on the fence’, or were negative about it, can see some merits and then they actually join in." *S2, Surgeon* (Not everyone has to agree) "Most parents do not want their child to have an operation, but that is the same for most children. But when you see a massive chunk and you’re like, my training and my experience tells me that I should fix it, then they get randomized to nonoperative and you see them at three months and they’re doing absolutely everything and you’re like, oh, okay, and so we’re all learning." *S20, Surgeon* (Desire to surgically fix the injury) "In surgical trials, it’s quite personal for a surgeon. Surgeons do know best, and to question that, as a member of the research staff, to say 'well this person meets the criteria for the study and so we should include them' is hard." *S18, Research Associate* (Hard to challenge surgeons)
**ii) Investing time and energy - how do we make it work?** Surgical staff and research associates invested time and energy integrating SCIENCE into daily practice so that the study worked in their clinical area. Staff found creating space, giving time, and developing a research pathway were key to recruitment. Creating space for research within busy clinical areas was challenging. Organizational knowledge, good relationships, dedicated research delivery expertise, and digital support facilitated this work.	"The children’s fracture clinic is a busy thing, children are booked in for five-minute slots during which you have to see, assess them. and do all the notes and things. So, it’s not an ideal recruiting ground and so sending them away was reasonable and sensible, and I think helpful." *S3, Surgeon* (Busy clinics) "We’re a bit stretched for space in our clinic, but we’ve got a counselling room which isn’t a clinical space - it’s just got a couch in there and so we tend to take them off into that space. So, we try and take them out of the clinic room so we’re not holding the clinic up and so there’s less pressure on the time then to get that done. So yes, it works well for us." *S18, Research Associate* (Not holding up clinic) "We didn’t have any problems. It was nice to have that time to be able to feel unhurried and unrushed. We’d definitely make sure that they [potential participants] were at the beginning of clinic again - the beginning or end of clinic, so that you have time." *S1, Research Associate* (Making time) "She had time to consider, well the family had time to consider the videos, the website at least during that weekend - so they were given that information." *S4, Research Associate* (Time to make sense of the information) "In terms of recruiting, I think it’s obviously massively helped having a supportive research nurse within our department. I don’t think it would have been possible to be involved with it without her input, because just all the form filling, keeping in on the right track, and keeping on top of future emails from your [trial] team that comes. If we didn’t have the research nurse support then there’s no way we would be able to do it." *S13, Surgeon* (Supportive research delivery staff) "If I am not there, one of them [the surgical team] would pick it up on my behalf, call the research nurse and a registrar, and say this is a fracture for the trial. They’d also make sure that I know about it. So, it is a team approach, I’m not on my own and I very much enjoy the help of my colleagues." S5, Surgeon (A team approach) "Everybody was so responsive and helpful [Trial team]. Whatever the IT [Information Technology] chap does to make this database so quick and easy and responsive is actually, really makes everything go very smoothly." *S1, Research Associate* (Responsive trial team) "They come with their preferences; some might come with their preference from home or maybe ED [emergency department]. We don’t know what’s been said, because they’ve been seen in ED, and so they’ve already been told which makes the study difficult to recruit to because they’ve already got that bias." *S17, Research Associat*e (Family have a preferred treatment)

**Table III.** Activities to facilitate recruitment from theme one.

Activities to facilitate recruitment from theme one are:
[Research Associates] Accept that individual surgeons have a preference for treatment of a paediatric medial epicondyle fracture, and work with this to enable recruitment [Surgeons] Consider the evidence for and value of the research question, your personal professional preference, and what is valued by the surgical community, to best enable recruitment [Surgeons] Continue ongoing reflection on treatment outcomes [All] Support teamwork and recruitment using the principle of community equipoise if personal equipoise is lacking [All] Work as a team with surgeons and research associates [All] Engage in the national online social media communities, to promote enthusiasm, overcome recruitment challenges, and refine position of equipoise All] Create a quiet family space away from the clinical area to best enable consent conversations [All] Use a tablet computer to enable family access to the study information and to sign the consent form [All] Allow time to facilitate family decision making [All] Develop a research pathway, for the family, tailored to local staffing and context.

### Theme two

**Enabling parental/child decision making:** Staff understood the challenging nature of the decision facing parents and children when offered the opportunity to participate in the study. They drew on family-centred practice to engage with children in a variety of ways with due consideration to the distress and maturity of the child

**i. Building trust and confidence. What can we do to help parents?**

Staff engendered family trust and confidence by understanding the parent/child experience, generating interest in the research study, sharing information, and supporting the family as they made their decision. Parents’ confidence to make a decision was challenged by the emergency context, the disparity in the magnitude of the two treatments (surgery or no surgery), and treatment uncertainty. Staff found it helped to understand the family’s experience of injury, anxiety, distress, and parental expectation that their child would receive the best treatment.

"I think the parents really struggle with the context, because they are coming to a doctor expecting to be told that this is what treatment you should have. I think when you say, 'Well actually, it could be this, or you could do this', they almost lose a little bit of confidence in you as their treating doctor." *S13, Surgeon*

Staff conveyed the importance of the research in finding out the best way to treat injured children and the exciting National and International context. Sharing information with consistent, clear, focused messages was considered crucial. Staff valued time to learn about parent/child experience of trial participation and reflect on ways of conveying trial information and use of language. Information from the Chief Investigator and trial team, along with opportunities for discussion with colleagues were considered helpful. Families were felt to respond well to staged study information, using the explainer video (carefully scripted animations explaining clinical trials and the SCIENCE study) and the study website information, in combination with shared family time. Staff felt it was important to ensure that attention was paid to both parents and child.

"A lot of children haven’t had that experience [of injury and treatment], and it’s that apprehension of not knowing. When you’re taking consent and giving the information, you have to gear it to two separate parties, almost at completely different levels. So, you’re trying to have a conversation with the child explaining it to them and making sure they understand, but then equally making sure the parents understand." *S12, Research Associate*

The disparity in magnitude between the two treatments, and potential complications, made explanations more complex. Family time was often required to talk about the study, followed by further questions and multiple interactions to fill in the gaps in understanding. Staff found families struggled with the decision to take part. They could have a strong preference for either treatment, choosing to avoid surgery and implant removal, or preferring operative fixation. Alternately, they did not know what to do.

"One particular conversation went round and round in circles within a family. They ended up so confused and then unhappy with any outcome, thinking that it wasn’t going to be the best treatment. I couldn’t advise that any treatment was better, as it was unclear in my mind. It just confused the family." *S16, Surgeon*

Families required differing levels of support to make their decision and staff used information to gently challenge fixed views of treatment. Information sharing and support were key elements that underpinned family trust in staff and confidence to make a decision. Staff were aware that, for families, the decision had psychosocial and practical considerations, and they needed to make sense of the study within a family context.

**ii. Engaging in flexible ways. How can we engage with children?**

Staff valued the child’s contribution within the principles of family-centred care and aimed to see family members together. They worked with families exploring preferred treatments, fears about surgery, and children’s thoughts and feelings about trial participation. A range of ways were used to communicate with children such as, play, drawing, and digital media using the tablet computer.

"The child, it’s their body, and they are the ones who are going to be submitted to an intervention - whether it’s surgery or no surgery. They are the ones who make the decision as well as the parents. It’s really important to involve the child, to engage with the child to get them comfortable with you, tailor the information to their level of their understanding, creating and building a rapport, and all of this takes time." *S4, Research Associate*

In the study, all children were aged under 16 years; therefore, parents provided consent for their child to take part. The child’s influence on decision making varied, dependent upon their level of understanding and their degree of pain and distress. Families often held a treatment preference, which could be for either treatment, but wishing to avoid surgery was most common. The unknown aspect of surgery created anxiety, and a cast was less disruptive to family life. Staff felt it was important to respect children’s wishes.

"The child today, a girl, was very firm. She was ten years and two months and was very confident in saying what was her opinion and what she wants. She was really firm and that was the end of the conversation." *S8, Research Associate*

Staff valued communicating with children in a way that was meaningful to them. They drew on education, prior experience, and knowledge of the school curriculum to inform their interaction. Assessing a child’s maturity was challenging, but younger children tended to take little part in the discussions. Some children were more mature than was expected.

"He was unusual because he was more mature than I was expecting and made a very balanced decision. There are some adults that are still childlike, and they never really mature and it’s just completely arbitrary. You can’t predict who it’s going to happen to and what their level of maturity is." *S2, Surgeon*

Interactions varied depending on the state of the child and their readiness to engage, but use of the tablet computer, activities involving play, certificates of participation, and the study information videos were well received. Staff found including children within family-centred care, working with preferences, and engaging with the child important strategies for facilitating participation in the SCIENCE trial. Table IV and Table V gives further supporting evidence for theme 2.

**Table IV.** Examples of participant quotes for theme two, 'enabling parental/child decision making'.

**Theme two, 'Enabling parental/child decision making'** Staff understood the challenging nature of the decision facing parents and children when offered the opportunity to participate in a trial. Family-centred practice and engaging with children in a variety of ways, with due consideration of their distress and maturity, were valued.	
**Category and definition**	**Examples of participants’ quotes with the codes in brackets**
**i) Building trust and confidence - what can we do to help parents?** Staff engendered family trust and confidence through: understanding the parent/child experience, generating interest in the research study, sharing information, and supporting the family as they made their decision. Parents’ confidence to make a decision was challenged by the emergency context, the different nature of the treatments (surgery or no surgery), and treatment uncertainty.	"[As a member of the public] The person you’ve got in your mind of the all-knowing doctor is saying 'we don’t know'. That, I would imagine, is quite an unnerving thing - because you expect them to know. They’re the doctor, they’re the surgeon, they’re the consultant - they know about this thing, this is their job – but, they’re saying well actually, we don’t." *S12, Research Associate* (Doctors know best) "When they’re here I tell them that the reason they’ve been brought is, that they have a very interesting fracture and there is a lot of excitement about it at the moment. It seems to get them a bit more on side. I tell them about the study and the reason behind the study, and that we were really hoping they would take part." S7, Surgeon (An exciting fracture) "It just takes a little bit more thought….because you’re not comparing two types of surgery, you’re not comparing two types of physiotherapy or anything, you’re comparing surgery with something that is very different." *S12, Research Associate* (Comparing two very different treatments) "As soon as you say 'the outcomes are good with both' they say, well 'we won’t have an operation', and that’s okay. We try and explore why that’s the case, but if they’re adamant that is what they want then that’s okay." *S18, Research Associate* (Avoid an operation) "Some are just completely bewildered by the full information sheet and it might put them off straight away. However, they’ve kind of got a feel for what the study is, through the video, and think ‘oh that sounds okay’. Then they read the information sheet and you’re more likely to get success." *S10, Research Associate* (Staging information giving) "The animation [video] didn’t help and my explanations didn’t help or helped but confused the family, but the Operation Ouch lads [children’s television medical personalities], when they saw that they said right, let’s do it." *S16, Surgeon* (Range of support tools) "She could not make up her mind. She was very confused. She could not make any decision, because she did not have any medical knowledge. So, she said 'why don’t I put my child in the trial and risk it, and ask the computer to decide on it', because she could not make a decision." *S8, Research Associate* (Struggle to make a decision) "Is one procedure going to be more painful than the other is also very important. 'How is my child going to go back to school and play sports?', 'Will it limit their interactions?'. There’s a lot of other questions that parents have concerns about and that are not quite addressed by the website or the videos." *S4, Research Associate* (Lots of questions)
**ii) Engaging in flexible ways - how can we engage with children?** Staff valued the child’s contribution within the principles of family-centred care and aimed to see family members together. They worked with families exploring preferred treatments, fears about surgery, and children’s thoughts and feelings about trial participation. A range of ways were used to communicate with children such as play, drawing, and digital media using the tablet computer.	"I think the material focused on children is really good and is very helpful. I have definitely seen it work to the extent that a child has said that they want to be in the study, rather more firmly than the parent. The parent says that’s fine, if you want to do it that’s fine. I think that is important and what we have works quite well." *S3, Surgeon* (Child friendly material) "The child was worried about not going to school because it was one of the last weeks [of school] and really wanted to be with their friends. [The child asked] 'Could the surgery be done at the weekend?', which would not have as much impact on the school activities. All of these activities have to be considered before you press randomize. We need to understand if we can negotiate all of these complex needs for patients, for clinicians, for whole families, for the whole structure - it’s also about relationships." *S4, Research Associate* (Listening to the child’s concerns) "I think for a lot of adult nurses, that are doing this [children’s study recruitment], unless they’ve got children at home themselves, that it is a very different process. It’s very different having a conversation with a seven-year-old, and trying to recruit [them] to research than it is having a conversation with a grown adult. I enjoy it, but equally I know people that – it’s not that they don’t enjoy it – but, they don’t have the confidence to do it, and they don’t feel comfortable doing it." *S12, Research Associate* (Confidence and skills to talk to children) "Some will say they want to go with the plaster and see how it goes, before they even think about it - because everybody’s scared of surgery. The children who are a sensible age, will not want surgery. Children who are younger and can understand an operation, start crying and screaming and having a fit." *S17, Research Associat*e (Children often don’t want surgery) "I think Dad was 'whatever' [slang for indifferent] and Mum was torturing herself about the whole thing. Then, they decided to go forward. The child was randomized to an operation and everybody was raging. Everybody was really unhappy about the situation then - Dad was, Mum was, the child was." *S6, Research Associate* (Strong emotions about surgical treatment) "It was quite interesting because the parent - the mum - had her own views on whether her child should go in the trial or not. But he [the son], was showing a bit more maturity and just turned round and said 'that sounds like a really good idea, I’d like to go in the trial, I’ve heard both sides of it'.” *S2, Surgeon* (Making their own decision) "The child was very nervous to start with, so I started by asking about the toy that the child had in her hand. So, we started engaging in conversation about the toy, then school and activities. Then the child asked me about if she could choose which treatment she could have. I explained that if she went into the trial, we try to make things fair and we had two very similar groups of children – where one group would have one treatment and the other group would have the other treatment. We wouldn’t be able to choose, we want to find out the treatment that is the best." *S4, Research Associate* (Engaging in conversation) "They might be sitting there colouring, or playing on their phone, but you know they’re listening to absolutely every single word that’s being said - even if it’s not being directed at them. I think some of the adult teams don’t always realise that." *S19, Research Associate* (Child can listen while playing)

**Table V.** Activities to facilitate recruitment from theme two.

Activities to facilitate recruitment from theme two are:
Keep communication direct, honest, and straightforward Understand that the preference of the family is for 'the best treatment for them' Tailor reassurance to parents’ and child’s experience of injury Generate excitement for the study, share the reasons why the study is important Present the study as a positive opportunity Use explanatory videos as an introduction to the study Share information using consistent language, and careful use of words Be aware that families vary in the amount of time required for decision making Avoid telling families the treatment plan prior to discussion about the study Inform all relevant family members at the same time Consider how to present the two different treatments Avoid conveying there is a choice between the two treatments, maybe inclusion in the study is the optimal approach Be aware of signs that parents feel pressured to make decisions Accept that some families find the decision very challenging, maybe frame the study as an opportunity to avoid a decision which even experts would be unable to make Engage with children as part of family-centred care Listen to children’s thoughts and feelings Be aware of the maturity of the child Use play and the tablet computer as ways of engaging with children Include children in the conversation even if they do not appear interested

## Discussion

The study identified the importance of 'leading through expertise' when recruiting to a study of a rare injury in paediatric orthopaedic trauma. Expert surgical and research delivery staff valued research evidence, personal commitment, relationships, and teamwork. Drawing on their expertise and these values, staff made the study work within existing resources and the organizational context, and facilitated parent/child participation by engaging with and supporting family decision making.

For the SCIENCE study, surgeons demonstrated leadership through the theme choosing ways of making it work and considered, is it a good question? Our study found that, for paediatric medial epicondyle fractures, surgeons put aside their strong personal treatment preferences to facilitate the generation of new evidence. Clinical expertise, knowledge of complications from both treatments, the need for evidence-based surgical practice, and community equipoise all supported commitment to the research question. Collegial support and reflection facilitated through a large WhatsApp community helped maintain a position of equipoise when surgeons felt challenged by the extremes of fracture displacement. Other trials have found equipoise challenging which can limit recruitment.^15-17^

In choosing ways of making it work staff also considered, how do we make it work? Expert leadership from research delivery staff was essential within busy clinical contexts. Unfaltering enthusiasm, often an important element of research cultures,^18^ was used to combat the rare occurrence of this injury and the organizational challenges of the COVID-19 pandemic. Lack of experience of research processes, combined with inconsistent digital infrastructure and insufficient space for research in clinical areas were everyday challenges for staff. Future research could consider how staff build expertise and sustain vibrant research cultures that enable recruitment to studies of rare injuries. Knowledge diffusion could be developed through national leadership roles in orthopaedic trauma for research associates. Garnering expertise in trauma research delivery could facilitate recruitment to trauma trials through networking, education, and mentoring of staff across countries.

Surgeons and research delivery staff demonstrated leadership through enabling parental/child decision-making and considered, what can we do to help parents? Our study found that clinical expertise was essential to support parents and children when making a decision about trial participation in an emergency context. Staff valued the provision of high-quality care and used their clinical knowledge and skills as the basis for engaging with families. Building trust and family confidence was facilitated through a holistic understanding of the patient group. Staff valued understanding the parent/child experience, sharing knowledge of treatment and research pathways, and skill in engaging with children. The disparity in magnitude between the two treatments (i.e. surgery or no surgery) was challenging for families. Staff aimed to build trust and confidence through the provision of honest balanced information, giving families time to assimilate the information and support for their decision making. These principles, valued by parents and staff,^7^ helped staff manage parental/child anxiety and the emotions generated by injury and place the study within the context of their daily life. The rarity of the injury was a barrier to recruitment. Staff education, a simple digital recruitment process, and staff enthusiasm compensated for the reduced learning opportunities that may have been gained through regular recruitment. Staff were aware of the balancing act, whereby one small action can influence family preference for treatment.^17^ The study information and website, explainer video, and reflection on use of language helped staff maintain a balanced approach to the study.

Surgeons and research delivery staff demonstrated leadership through enabling parental/child decision making and considered, how can we engage with children? The degree to which children were involved or protected from decision making was dependent on the maturity of the child and decision-making style of the family. Further research, with parents/children and the development of educational tools, may help to support family decision making in an emergency context. Building trust and family confidence in research may help to ensure parents and children have a good experience of research and embed research within daily home and clinical life.

The study identified staff experience of ‘leadership through expertise’ providing direction for future studies of rare paediatric traumatic injury. The purposive sample of staff included surgeons and research delivery staff from 18 NHS hospital sites geographically spread across the UK. A range of experiences were obtained to ensure different ways of working were represented. Clinical, research staff, and patient and public involvement and engagement (PPIE) partners at presentations show resonance with the findings of this study. Staff from sites that were unable to take part in the study may have provided further experience of the barriers to research in children with rare injuries.

In conclusion, for this rare injury in paediatric trauma, ‘leadership through expertise’ was critical for successful trial recruitment. National and local surgical networks, facilitated through technology, were critical to support trial activity. Similar national leadership for research delivery staff could facilitate knowledge diffusion and research pathway development. Parental/child trust and confidence to make a decision about trial participation could be enhanced by further work focused on children’s engagement with research. Educational tools that engage a child may help to support family decision-making in an emergency context.

**Take home message**

- Recruitment to this study of epicondyle fractures in children was achieved through national and local leadership with a strong foundation in clinical expertise.

- Strong treatment preferences were put aside though a commitment to answer the research question.

- Teamwork, enthusiasm, personal commitment, organizational knowledge, and the ability to understand the family experience of injury and research were essential for building parental/child trust and confidence.

## Data Availability

The data that supports the findings are available to other researchers from the corresponding author upon reasonable request.
